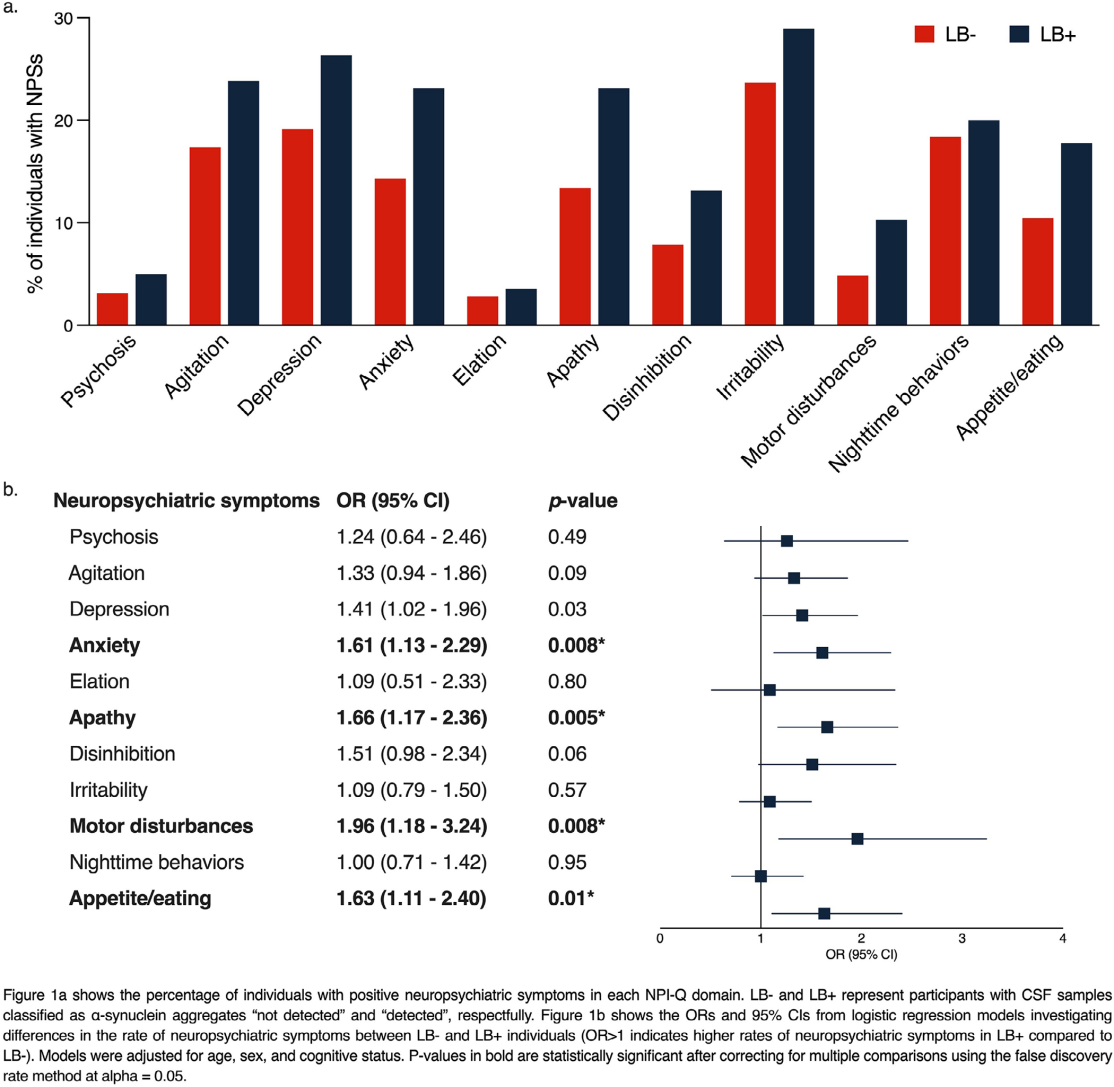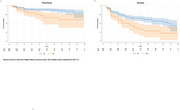# Effects of in vivo Lewy body pathology on neuropsychiatric symptoms across the Alzheimer's disease continuum

**DOI:** 10.1002/alz70857_106523

**Published:** 2025-12-26

**Authors:** Douglas Teixeira Leffa, Guilherme Povala, Pamela C.L. Ferreira, João Pedro Ferrari‐Souza, Guilherme Bauer‐Negrini, Matheus Scarpatto Rodrigues, Livia Amaral, Firoza Z Lussier, Marina Scop Medeiros, Carolina Soares, Arthur C. Macedo, Joseph Therriault, Pedro Rosa‐Neto, Dana L Tudorascu, Eduardo R. Zimmer, Bruna Bellaver, Tharick A Pascoal

**Affiliations:** ^1^ University of Pittsburgh, Pittsburgh, PA, USA; ^2^ Universidade Federal do Rio Grande do Sul, Porto Alegre, RS, Brazil; ^3^ McGill University, Montreal, QC, Canada; ^4^ Universidade Federal do Rio Grande do Sul, Porto Alegre, Rio Grande do Sul, Brazil

## Abstract

**Background:**

In Alzheimer's disease (AD) dementia, co‐pathology is frequently observed, with Lewy body (LB) pathology among the most common findings. Post‐mortem studies support a higher frequency of neuropsychiatric symptoms in individuals with AD and LB co‐pathology. To date, however, the effects of LB pathology measured in vivo on neuropsychiatric symptoms in AD is underexplored. Here, we evaluated the cross‐sectional and longitudinal effects of in vivo measured LB pathology on neuropsychiatric symptoms across the AD continuum.

**Method:**

We analyzed data from a total of 1,169 individuals from ADNI (426 cognitively unimpaired and 743 cognitively impaired; 47.13% women; mean age of 73.05 years). All participants had baseline in vivo LB pathology measured through CSF alpha‐synuclein seed amplification assays, as well as neuropsychiatric assessments using the NPI‐Q for up to 10 years. A subgroup of 977 participants had baseline CSF amyloid‐β (Aβ_1‐42_) and *p*‐tau_181_ levels. We explored cross‐sectional and longitudinal effects of LB on neuropsychiatric symptoms using logistic regression and Cox proportional hazard regression models, respectively. Analyses were controlled for age, sex, and cognitive status. We further evaluated the independent effects of LB, Aβ and tau by including all three pathologies in the same model.

**Result:**

In cross‐sectional analyses at baseline, the presence of LB pathology was associated with higher rates of anxiety (OR=1.61, 95% CI=1.13‐2.29, *p*‐value=0.008), apathy (OR=1.66, 95% CI=1.17‐2.36, *p*‐value=0.008), motor disturbances (OR=1.96, 95% CI=1.18‐3.24, *p*‐value=0.008), and appetite disturbances (OR=1.63, 95% CI=1.11‐2.40, *p*‐value=0.01) after correcting for multiple comparisons (Figure 1). In longitudinal analyses, the presence of LB pathology was associated with a higher risk of developing psychosis (HR=2.15, 95% CI=1.30‐3.56, *p*‐value=0.003) and anxiety (HR=1.70, 95% CI=1.22‐2.36, *p*‐value=0.001, Figure 2). The cross‐sectional and longitudinal effects of LB were independent of Aβ or tau.

**Conclusion:**

Our results suggest that in vivo‐measured LB pathology is associated with a higher frequency of neuropsychiatric symptoms, with apathy, motor disturbances, appetite changes, anxiety, and psychosis as potential early neuropsychiatric manifestations of LB pathology in individuals across the AD continuum. These findings underscore the potential of in vivo LB detection as a marker for identifying individuals at elevated risk of neuropsychiatric symptoms, both in clinical trials and in clinical practice.